# Development, Implementation, and Evaluation of a Structured Reporting Web Tool for Abdominal Aortic Aneurysms

**DOI:** 10.2196/resprot.2417

**Published:** 2013-08-16

**Authors:** Sulafa Karim, Christian Fegeler, Dittmar Boeckler, Lawrence H Schwartz, Hans-Ulrich Kauczor, Hendrik von Tengg-Kobligk

**Affiliations:** ^1^German Cancer Research CenterDepartment of RadiologyHeidelbergGermany; ^2^University of HeilbronnFaculty for InformaticsHeilbronnGermany; ^3^University Hospital HeidelbergDepartment of Vascular and Endovascular SurgeryHeidelbergGermany; ^4^Columbia UniversityDepartment of RadiologyNew York, NYUnited States; ^5^University Hospital HeidelbergDepartment of Diagnostic and Interventional RadiologyHeidelbergGermany; ^6^University Hospital of Bern, InselspitalUniversity Institute of Diagnostic, Interventional and Pediatric Radiology (DIPR)BernSwitzerland

**Keywords:** radiology, structured reporting, vascular surgery, abdominal aortic aneurysms

## Abstract

**Background:**

The majority of radiological reports are lacking a standard structure. Even within a specialized area of radiology, each report has its individual structure with regards to details and order, often containing too much of non-relevant information the referring physician is not interested in. For gathering relevant clinical key parameters in an efficient way or to support long-term therapy monitoring, structured reporting might be advantageous.

**Objective:**

Despite of new technologies in medical information systems, medical reporting is still not dynamic. To improve the quality of communication in radiology reports, a new structured reporting system was developed for abdominal aortic aneurysms (AAA), intended to enhance professional communication by providing the pertinent clinical information in a predefined standard.

**Methods:**

Actual state analysis was performed within the departments of radiology and vascular surgery by developing a Technology Acceptance Model. The SWOT (strengths, weaknesses, opportunities, and threats) analysis focused on optimization of the radiology reporting of patients with AAA. Definition of clinical parameters was achieved by interviewing experienced clinicians in radiology and vascular surgery. For evaluation, a focus group (4 radiologists) looked at the reports of 16 patients. The usability and reliability of the method was validated in a real-world test environment in the field of radiology.

**Results:**

A Web-based application for radiological “structured reporting” (SR) was successfully standardized for AAA. Its organization comprises three main categories: characteristics of pathology and adjacent anatomy, measurements, and additional findings. Using different graphical widgets (eg, drop-down menus) in each category facilitate predefined data entries. Measurement parameters shown in a diagram can be defined for clinical monitoring and be adducted for quick adjudications. Figures for optional use to guide and standardize the reporting are embedded. Analysis of variance shows decreased average time required with SR to obtain a radiological report compared to free-text reporting (*P*=.0001). Questionnaire responses confirm a high acceptance rate by the user.

**Conclusions:**

The new SR system may support efficient radiological reporting for initial diagnosis and follow-up for AAA. Perceived advantages of our SR platform are ease of use, which may lead to more accurate decision support. The new system is open to communicate not only with clinical partners but also with Radiology Information and Hospital Information Systems.

## Introduction

The report is one of the most essential components of the daily work for a radiologist. It is the way that the radiologist communicates with referring clinicians and includes diagnostic findings, conclusions, and sometimes recommendations. The written radiology report serves as the primary mode of communication for physicians in the diagnostic everyday work and is an invariably part in the health record of a patient. In the last decade, radiological reports consisted of typed, dictated, or even handwritten text [1].

While there have been some advances in the development of reporting software systems, radiology reports generally are lacking in structure, clarity, conciseness, consistency, and readability [2]. Follow-up reporting of similar radiology exams are frequently different from the baseline studies and are often confusing due to the lack of similar structure. In spite of the critical importance of radiology reports, they consist mostly of one large text module, they are non standardized, maybe incomplete, and unclear [3,4]. The use of free-text, conventional dictation allows radiologists to dictate in narrative style and in any level of detail. Frequently, if two radiologists are reporting the same imaging data, the final report may look very different; even there is an equal understanding about all of the radiologic findings and conclusions. Thus, it is almost a challenge for physicians to interpret and analyze radiological findings accurately and efficiently. Some improvement was made by introducing structured text modules which are reached in consensus within an individual radiology department or even suggestions of international Radiological Societies, (eg, the RSNA). These text modules are especially helpful for reports without pathological findings or simple reports. By reducing the possibility of variability in other areas within medicine, quality management has been improved [5]. Likely, there may be an improvement in quality in radiological reports by less variability [6].

To improve clinical acceptance of our radiology reports, the involved departments decided to analyze the need for more structure and, particularly, standardization.

This pilot study embraces creation and implementation of a software application for Structured Reporting (SR) for abdominal aortic aneurysm (AAA). The objective of the development of a SR application was to gain an efficient comprehensive reporting standard for AAA. An additional aim is to introduce a novel method that provides efficient medical reporting with less possibility for error among expert and non-expert readers.

## Methods

### Participants

To understand the key data of this implementation, we conducted interviews of hospital staff within the departments of vascular surgery (senior physicians, n=2–at least five years work experience; residents, n=2–at least two years work experience) and radiology (senior physicians, n=2–at least five years work experience; residents, n=2 at least one year work experience), supplemented by a review of project documentation. We analyzed qualitative data from field observations and formal interviews. Interviews took place during a 1-year period following system implementation and were performed by the same interviewer. The investigator recorded notes during the consultation hours. Interview notes were iteratively reviewed to identify the most common and essential elements for the chosen local clinical environment. Subsequently, the usability of the introduced method is assessed by evaluating its acceptability by the users based on questionnaire responses.

### Actual State Analysis

Process orientation is one of the most vital elements of quality management. To optimize and devise workflow processes more efficiently, it is necessary to reflect the whole cycle of interactions and different issues within an organization [7]. To increase shared knowledge and improve the processes related to radiological reporting, we proposed a pilot study using the Business Process Modeling Notation, a user-oriented language for a model of the specifying business processes [8]. The objective of this process was to analyze decision making within the departments of vascular surgery and radiology of a multidisciplinary working group. Previous experiences using this notation in process modeling within exactly those departments are not known.

**Figure 1:**
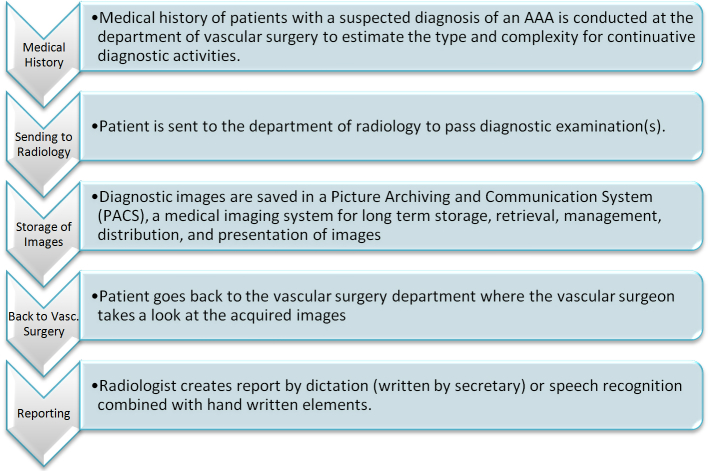
Related processes between departments of radiology and vascular surgery.

A hospital workflow consists of a sequence of connected steps, a sequence of operations, declared as the work of a person [9]. In terms of treatment of an AAA vascular surgeons request computed tomography (CT) images to have reliable measures of selected positions of the aorta. As a result of the increasing life expectancy, AAA is one of the most common atherosclerotic arterial diseases involving the aorta [10]. Diagnostic investigation, quick therapy plans, post-operative control, and follow-up management are common time points of interaction between the involved clinical departments. The related processes can be seen in Figure 1.

### SWOT Analysis

#### Overview

The SWOT analysis is a strategic planning method. It aims to identify the strengths, weaknesses, opportunities, and threats of a project [11]. Table 1 is a SWOT analysis of the pre situation within the departments of radiology and vascular surgery. The analysis was performed to evaluate the use of free-text reports approach to develop a new concept of reporting. Attributes of the departments that were helpful to achieve the objective were defined as strengths; attributes considered detrimental for our purpose were defined as weaknesses. Additionally, external conditions considered as helpful to achieve the objective were defined as opportunities. External conditions that could be detrimental to the objective were defined as threats [12].

#### Strengths

The most relevant strength found was that the department of radiology has a good technical infrastructure and a well coordinated IT management with an optimal support for user and workflow. All clinical information can be fetched at any time and from every workstation.

#### Weaknesses

The current free-text reports do not fulfill the daily expectations of the in-house clinical partners. The reports were individually unstructured containing a monolithic text module. A quick overview of defined medical parameters was not ensured (eg, a direct comparison to former reporting results). The clinician was missing a structured form and essential information in one view. There was no trust that measurements were taken correctly by radiologists.

#### Opportunities

The adoption of a structured, standardized reporting provides the potential for a greater chance of acceptance by referring clinicians. For clarity: the readers know where they can expect the description of a certain detail or aspect within the report. By gaining acceptance by all users in the conception of a new method of reporting, individual user requirements can be considered. According to the collaboration of both departments, the essential medical parameters can be discussed and implemented. The quality of a diagnosis of a clinician could be ameliorated by a second opinion by radiologists. Hereby, the communication could be enhanced between the clinical departments and an optimization of the processes can be achieved. Another advantage of structured reports is that clinical data and measures can be used for scientific and statistical needs.

#### Threats

A risk of the implementation of such a system is that radiologists may feel that they were forced to use a system which they do not agree with and will refuse to use it clinically. It may take longer to fill in a standardized report at first use compared to the creation of individual text. Another risk is that it might be inadequate for individualized disease conditions or differential diagnosis. Standardized reporting tools could also be regarded as detrimental to “personalized medicine”.

**Table 1:** SWOT analysis performed between departments of radiology and vascular surgery.

	Descriptions	Strengths	Weaknesses
**Opportunities**			
	Optimization of clinical workflow	Technical infrastructure (HIS^a^, RIS^b^, and PACS^c^, modalities)	Communication to vascular surgery
	Quality improvement by second opinion, professional expertise (two medical specialists)	High standardization of routine workflow	Involvement of specialized requests of vascular surgeons in radiological reports
		Specialization within team	Time requirement
		High quality mangement	Unstructured format of radiological reports (free text)
**Threats**			
	Radiological reports do not fulfill formal expectations	Interface between radiology and vascular surgery	Improvement of quality of internal processes
	Not in due time availability of radiological reports	Specification of required topics	Development of structured radiological reports
			Bundling of expertise

^a^hospital information system.

^b^radiological information system.

^c^picture archiving and communication system.

### Technology Acceptance Model

Davis et al [13] first introduced the Technology Acceptance Model, which consists of four primary factors: external variables, perceived usefulness, perceived ease of use, and intention to use. Within this pilot study, we examined the actual acceptance of free-text reports by vascular surgeons at our medical center.

The structure of the report as well as its acceptance by the users, were the criteria we took as a basis [14]. Examining the external variables, the acceptance of free-text reports by our clinicians was assessed. Clinicians can be seen as customers and radiologists as suppliers in the value chain (ie, they are the primary stakeholders in data extraction). In our case, customer needs are important in the internal sense.

Since the radiologist defines the content of the report, there might be a mismatch regarding parameters expected by the surgeon (eg, in case of therapy planning or monitoring). A report usually starts by mentioning if prior exams (in-house or external) are present for comparison with date and modality. In general, a radiologist reports all pathological findings. Some reports strictly follow a topographical order, while others report relevant findings first than the less relevant ones depending on the “individual style” of the radiologist. A summary usually points out the relevant findings often in a hierarchical order and should contain a clinical interpretation.

A type of “visual clarity” of reports is usually missing since reports contain only one text module with all aspects and a short summary. The time needed to create a report varies with experience of the radiologist, his eagerness to describe every detail, and the reporting system including the technical skills of the radiologist to navigate the system. Finally, a report may need to be reviewed by a second colleague depending on the departmental rules and the experience of the first reader. Therefore, availability of a radiological report after the patient is coming back to the referring physician from a diagnostic examination is not guaranteed.

Perceived usefulness within state-of-the-art reporting is not given because clinicians might not read them due to their unstructured und unclear text modules as well as low quality in their constitution. Perceived ease of use cannot be declared as simplicity. The current reports are not always available in time and a quick overview about certain measures is not possible. Clinicians deduce their handling like individuals from their own expertise, knowledge, experiences, and perceptions without regarding the reports of radiologists.

The intent to use radiological reports is to obtain a second opinion of another qualified person, in this case from radiologists. On this note, clinicians are able to make an accurate diagnosis by consulting a radiologist. In this case, the best possible medical care could be assured.

### Determination of Medical Parameters

Prior to implementing a Web-based application for structured standardized reporting, we defined our internal medical reporting parameters of AAA by analyzing the existing workflow and requested report features based on interviews. To create a basis for efficient communication exchange between departments, it is necessary to agree on the most relevant medical parameters of AAA.

We reviewed existing literature about the classification and reporting standards of AAA [15]. The structured report conveys the relevant medical parameters in a logical manner to make it easy for clinicians to find the necessary data quickly.

### Usability Test: Statistical Analysis

Usability and reliability of the method is validated within a real test environment in the department of radiology. The prototype was used by a special focus group of radiologists. Real patient data (pseudonymised) related to AAA were first read using the traditional way of reporting, and then using the new implemented Web-based software tool. To avoid a bias, time between the two reporting methods was at least one week. For a comparison between both ways of reporting, analysis of variance (ANOVA) was used to show difference of time effort. A *P* value of less than .05 is considered to be statistically significant.

By evaluating its acceptability by the users, questionnaire responses were gathered to conduct a controlled comparison of the performance of the new software in relation to the medical transcriptionist way of reporting.

## Results

### System Design

The highly adaptive design of SR led to a variety of methods of its use. Our application consists of four layers; Layer I: as a database, we used the relational database management system MySQL (Oracle corporation, Redwood Shores, California, USA). Layer II: the entire database connection is encapsulated in the data access layer using Hibernate (Red Hat, Raleigh, North Carolina, USA). Layer III: the business logic layer is implemented with Spring (SpringSource/VMWare, Palo Alto, California, USA). Layer IV: the graphical user interface (GUI) is written in Extended Google Web Toolkit (GWT), an open source set of tools for implementing Web applications. By using the combination of Spring and Hibernate, the GUI and database can be easily replaced by other possibilities. The GWT (Google, Menlo Park, California, USA) was chosen because of the high graphical attractiveness for the user [16].

### Graphical User Interface

The SR offers several of the following advantageous features: the parameters included standardized point-and-click menu topics, including anatomy, measures, and additional diagnostic findings, listed by organ and dedicated pathologies. The whole application is structured into three tabs: Tab A: characteristics of pathology and adjacent anatomy, Tab B: measurements, and Tab C: additional findings including a free-text option for personal judgment. Clicking on one of these tabs presents predefined standardized options that can be chosen. The selection of the medical parameters is effected dynamically. In relation to referred clicks, the relevant parameters are automatically displayed to minimize the depth of the graphical interface. The use of free-text is restricted to a minimum, as the most relevant information is entered through user-friendly tab menus. Radiologists are also allowed to interrupt their report. All registered parameters can be saved. Furthermore, SR offers the functionality of generating a PDF file for a medical report. An export function for statistic reasons is also available.

### Characteristics of Aortic Pathology

#### Scope

**Figure 2:**
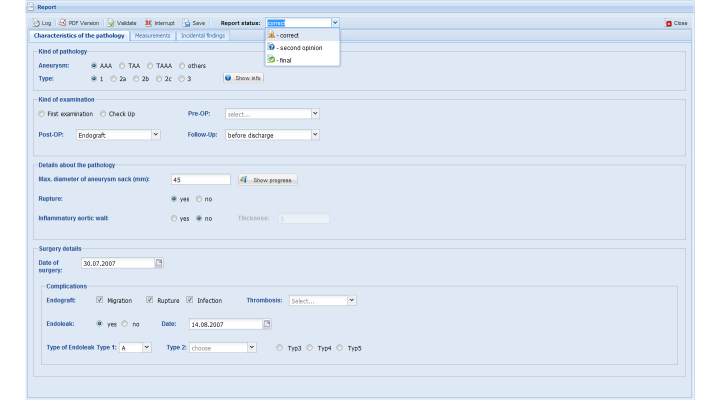
Characteristics of aortic pathology, including kind of pathology, kind of examination, details about the pathology, and surgery details with potential complications.

The scope of “characteristics of pathology” shown in Figure 2 constitutes of four items: (1) kind of pathology, (2) examination, (3) details about aortic pathology, and (4) details about surgery and potential complications. The options for the first item are: AAA, thoracic aortic aneurysm, and thoracic-abdominal aortic aneurysm. In the future it is planned to standardize radiological reporting for other aneurysms so that the template would change dynamically by clicking the required kind. The definition of the pathology requires defining the type of aneurysm (eg, Type I according to jointly used classification).

**Figure 3:**
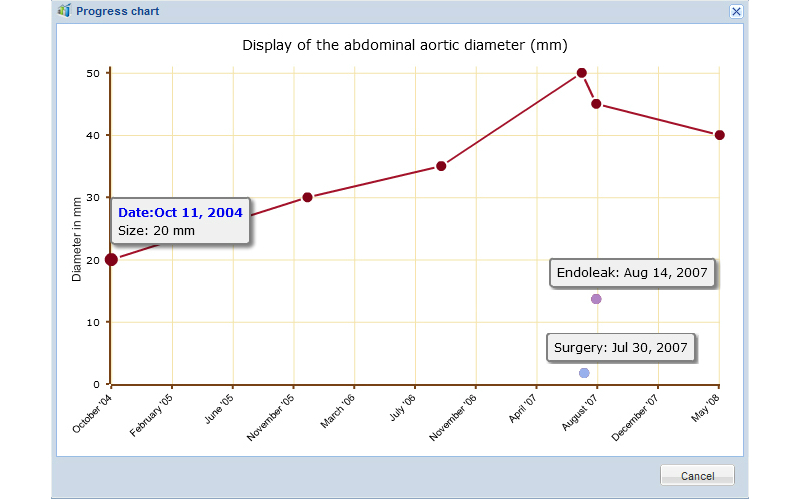
Example of a graph for progress-monitoring for an AAA, including the respective dates of the certain aortic diameter, surgeries, and endoleaks.

The options of “kind of examination” are: (1) first examination, (2) progress control, (3) Pre-OP: internal images, or external images, and (4) Post-OP: control, before discharge, 3 months, 6 months, 12 months, and 24 months. The radiologist continues with entering the measurement of the maximum diameter of the aneurysm sac. At this point, there is a possibility for clinical monitoring: all previously entered measurements in further radiological reports according to one patient are represented automatically in a progress diagram to have an overview about the whole development of the clinical history (Figure 3): automated monitoring for disease becomes possible through data mining. Dates of surgeries and complications, like an endoleak, are also embedded. Subsequently, the existence of a rupture or inflammation of the aortic wall can be noted. If post-surgery and Endograft for “kind of examination” certain additional information are needed: An Endograft can be migrated, broken, infectious, or have an intraluminal or extraluminal thrombosis. Furthermore, the occurrence of an endoleak with date as well as type (type 1-5) is necessary.

#### Measurement

**Figure 4:**
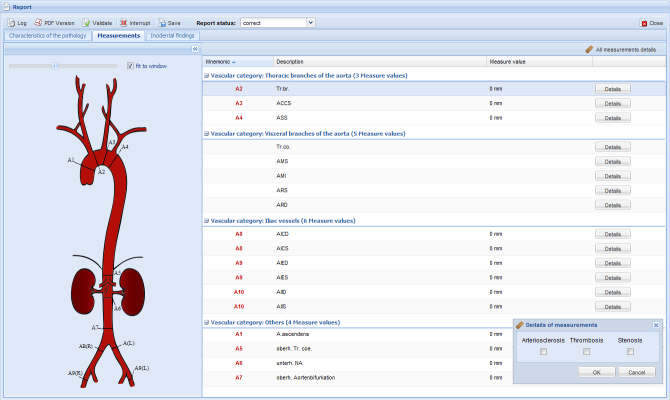
A1-A7 measurement positions depend on individual pathology. A8-A10 request the minimum diameter in case of stenosis or maximum in case of aneurysma.

The most relevant anatomic measurements of vessels along the aorta and its branches can be entered in the SR template (Figure 4). The position of each measurement is highlighted in a graphic as a sort of a guideline with the background to support slightly uncertain radiologists, for example residents (this input process also has a learning effect). Detailed information about the relevance of arteriosclerosis, thrombosis, or stenosis can be provided. The whole table is structured into (1) thoracic branches, (2) visceral branches, (3) iliac run-offs, and (4) miscellaneous. Regarding the required parameters, not every item requires a value or detailed information. Point-and-click choices were chosen to be inclusive of all commonly used parameters to describe aortic anatomy in detail.

#### Additional Findings

There is an option for radiologists to use free-text in the last tab of the report to have the opportunity to give additional relevant information if necessary. The free-text function allows writing individual medical advices or investigations that are not listed in the standard reporting.

**Figure 5:**
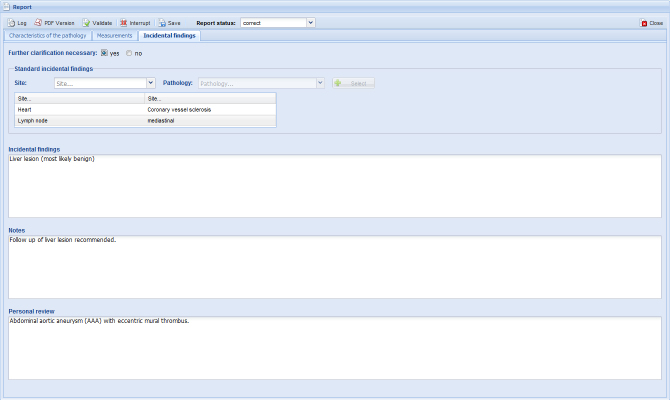
Additional findings and free-text options, including a list of standard incidental findings and free-text options for additional information of incidental findings, notes, and a concluding personal review.

The necessity of findings may need clarification with other clinicians, which can be checked at the beginning of tab 3 (Figure 5). Further, an alphabetically sorted list of additional findings is proposed which offers the main affected organs and their dedicated pathologies. The choices from the drop-down menus of an organ would result in complete, standard pathologies being created automatically. For example, the user could choose “lung,” and the pathology “embolism” would consistently appear in the report. Not all information can be captured in a structured report. A radiologist will need a narrative option to express unusual elements or to describe image parts that should also be documented. Such a function for more information of additional findings and a general conclusion can be entered in each free-text field in form of keyboard entry or speech recognition. Finally, a radiological conclusion should be entered.

**Figure 6:**
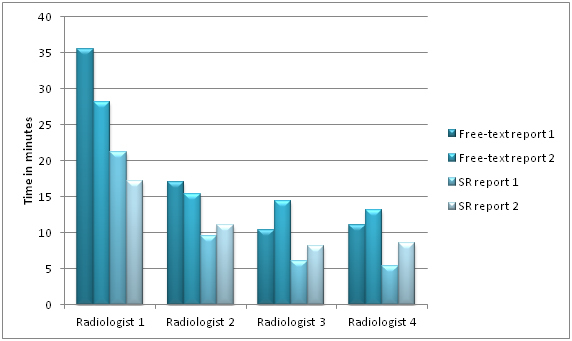
Comparison of time used for conventional versus Web-based reporting.

### Evaluation for Usability

The 16 CT images of patients with suspected as well as with confirmed diagnosis of AAA were reported twice by each reader (n=4). In this case, we ensured that reports were written with a one week time difference to avoid reader bias. Perceived advantages of SR are facilitation of workflows and ergonomics of the radiologist. The process of the usability test spread out in 2 scenarios:

1. Phase 1: Observation of radiologists during conventional reporting.
2. Phase 2: Observation of radiologists during SR.

In a routine clinical environment, a SR template can be used to generate uniform and consistent reports. Basic medical parameters of pathology should be entered, but it is also exempted to radiologists to report in a free-text area, especially for additional findings. The performance of structured reporting within the usability test is measured against the measured reporting times. By this way we can ensure objectively if there is an improvement versus the conventional way of reporting.

The free-text report is not dictated but rather typed in manually not to show a bias between the two reporting methods. The results for averaged reading time are summarized in Table 2.

To obtain a radiological report, ANOVA shows a reduced reporting time with SR compared to free-text reporting (*P*=.0001).

After several interviews, in-house referring clinicians and radiologists stated that structured reports had better content and greater clarity than conventional reports.

The evaluation procedure was based on DIN EN ISO 9241 software ergonomics in information technology and software product evaluation. The objective was to validate the usability and ergonomics of our new tool for SR. To evaluate the system’s effectiveness and efficiency, radiologists (2 senior physicians and 2 residents) were asked to complete a questionnaire using a scale from 1 (not useful) to 7 (excellent). The questions included 7 ergonomic principles, which are listed in Table 3 including mean values.

The results show that radiologists characterized the SR system as an innovative concept providing added value to the current reporting workflow (average rating=6.4/7.0).

**Table 2:** The time utilized for structured reporting and free-text option.

Radiologist	Free-text reporting [min : s]		Structured Reporting [min : s]		Mean Difference
	Read 1	Read 2	Read 1	Read 2	
1	36:51	28:22	21:15	17:23	13:18
2	17:07	15:33	9:57	11:09	05:47
3	10:37	14:46	6:06	8:11	05:33
4	11:03	13:14	5:41	8:58	04:49

**Table 3:** Ergonomic principles for software evaluation.

Principle	Mean
Adequacy of tasks	6.60
Self-descriptiveness	6.35
Expectation compliance	6.85
Controllability	7.00
Individualizing options	4.05
Learnability	7.00
Fault tolerance	6.85

## Discussion

### Summary

The current clinical workflow of radiological reporting and reading the report by the referring clinician offers room for improvement, and has been criticized by many groups [17,18,19]. We focused this workflow analysis and improvement on clinical communication between the departments of radiology and vascular surgery, and selected a common diagnosis: AAA. Besides time needed for the reporting processes, the reader experience during the reporting process plays important part. The quality of the reporting may be improved but it also may worsen as shown by various groups [20]. As we have seen in the evaluation, a standardized reporting scheme of suspicious findings on AAA leads to shorter reporting time rates than the standard random reporting.

### Application for Stuctured Reporting

Our Web-based SR application focuses on minimizing production time and improving the content of radiological reports for AAA. Clinicians can easily and quickly identify required data from radiological standardized reports. The SR can provide clinicians with a visual attractive interface showing the necessary measures to assist in quick decision making.

An important advantage for radiologists, especially for residents, is the new guided reporting method. Additionally, there is an opportunity to compare directly initial reports with follow-up reports, which is attractive for radiologists as well as for clinicians, respectively.

The system is flexible enough to incorporate various medical parameters which can be implemented easily. For a successful communication, it is vital to have a consensus concerning the content, meaning, technical expression, and medical terminology. By involving the users in the design process, we prevented the use of nonessential parameters. Our Web-application improves the availability of clinical information in the system (eg, provided by the requesting clinician during their request for radiological examination). By observing and interviewing the referring clinicians in the Vascular Surgery department, we found that SR in clinical practice may facilitate the professional workflow in several ways. First, it is possible to gain a more optimal understanding about relevant medical features in consideration of specific pathologic states. The clinicians know where they can find the requested information in a standard template. An inconvenient search in unstructured and unclear free-text is prevented. Particularly standardization through structuring of reports is a worthy goal at the temporal efficiency level. Emergency department encounter forms with a structured format have been shown to improve documentation and decrease test use compared with free-text recording [21].

Standardization of the reporting process is also beneficial for radiologists and enables (1) clarity of radiological reports, (2) quick creation of a radiological report, (3) temporal efficiency by radiological reports in time, and (4) capture of information for retrieval and reuses. The effort for a radiologist at the beginning of using a new reporting system is higher since they must first get used to the new type of radiological reporting. Our findings are auxiliary of the validity of recently proposed objections to SR in radiology [22]. Other groups have shown solutions for SR, especially in gastrointestinal endoscopy. Such applications have been developed, used, and also associated with accurate and complete data entry. The improvement and completeness of structured reports compared with free-text reporting are proved [23,24]. For pelvic ultrasound, a structured data entry system named UltraSTAR has been implemented to reach a high satisfaction among radiologists and gain slightly more complete but certainly more structured data than free-text reports [25].

In our introduced SR system, SR additional lab values can be implemented easily. It is a new era of smart reporting radiological findings with clinical information and the capability to add more clinical parameters if necessary. The goal to minimize the number of conscious steps which are necessary for report creation is achieved. The classification we have proposed in our implementation has the advantage that we reduced the medical parameters for AAA to a minimum in order to have a quick overview. Standardized reports also facilitate the use of real-time diagnosis and decision-making. Within radiology, SR has been most successful in mammography, where Breast Imaging Recording and Data System have been developed by the American College of Radiology [26]. Structured reporting has also been pursued in abdominal ultrasonography and was found to be a viable alternative to free-text dictation in terms of completeness, time efficiency, and user acceptance [27].

The presented SR platform can serve as a communication basis with hospital information systems (HIS), gathering all the necessary information (eg, the surgery date can be retrieved directly). In the initial pilot project, this functionality is integrated to demonstrate this relevant added value. In fact, the evaluated SR platform is a stand-alone version, but with wide-open interfaces, it can be easily adopted to several other systems. It enables context-specific clinical information retrieval from an integrated HIS and the related medical record. Since the presented SR platform is able to communicate via its open interface with other information systems, we embedded our tool as an external program into the existing RIS of our hospital to conduct the evaluation.

 The Aspect of An Open Interface Is Essential for Further Developments of Sr Systems to Build a Basis of Efficient Communication Using All Available Electronic Patient Records.

### Comparison to Free-Text Reporting

Compared with structured standardized reporting, free-text reporting implicates the following issues:

1. It is not ensured that all relevant clinical parameters are completely listed in a free-text report.
2. If values are missing within the report, the reader does not know if the measurement was not conducted.
3. Free-text-reports cannot be evaluated automatically in a systematic way.
4. Two radiologists produce two different reports that look very different in their order as well as in their number of details; even if there is an agreement of all findings and the conclusion. Thus, a direct comparison of two reports is nearly impossible.

The inherent disadvantages of free-text-reporting may be due to workflow differences, selection bias, and lack of uniform reporting standards as well as inaccurate reporting. For this reason, existing data of AAA reports are difficult to interpret in a systematic way for statistical purposes. Additionally, the terminology used to describe the findings is not universally agreed and often inconsistent. A conflicting consensus on the definition of a clinically stable patient is also prevalent [28,29].

Recent publications show that regarding the content of the reports, a SR system may also inherent disadvantages like causing a lack of curiosity of unexpected findings [30]. However, during this pilot study, the focus was not on the quality of content of the produced reporting.

### Conclusion

One important goal of using a new standard is to reduce the overall reporting time for radiologists as well as to achieve a faster and easier interpretation for referring clinicians. The presented new pilot application for AAA reporting facilitates the workflow for the radiologist and the referring physician. Clinical research may also benefit from SR since data can be extracted more easily for statistical analysis. Further studies are warranted to evaluate the quality of content using SR.

Structured standardized reporting has the potential to improve the patient care process and expand in other clinical realms within vascular imaging and other diseases. Current reports lack the structure to monitor disease in long-term follow-up patients having AAA. On this account, referring physicians prefer standardized reports because specific medical parameters can be extracted more easily than in a free-text report.

## Abbreviations

AAA: abdominal aortic aneurysm
ANOVA: analysis of variance
CT: computed tomography
GUI: graphical user interface
GWT: Google Web Toolkit
HIS: hospital information systems
RIS: radiological information systems
SR: structured reporting
SWOT: strengths, weaknesses, opportunities, and threats
